# Species Variety, Antibiotic Susceptibility Patterns and Prevalence of Enterotoxin Genes in Staphylococci Isolated from Foodstuff in Central Iran

**Published:** 2020-01

**Authors:** Laleh HOVEIDA, Behrooz ATAEI, Nour AMIRMOZAFARI, Zahra NOORMOHAMMADI

**Affiliations:** 1 Department of Biology, Science and Research Branch, Islamic Azad University, Tehran, Iran; 2 Infectious Diseases and Tropical Medicine Research Center, Isfahan University of Medical Sciences, Isfahan, Iran; 3 Department of Microbiology, School of Medicine, Iran University of Medical Sciences, Tehran, Iran; 4 Department of Biology, Science and Research Branch, Islamic Azad University, Tehran, Iran

**Keywords:** Staphylococcal food poisoning, Antibiotic resistance, Sequence analysis, Enterotoxins

## Abstract

**Background:**

The presence and diversity of *Staphylococcus* species and their enterotoxin-encoding genes in foodstuffs have not been comprehensively studied in some developing countries. This study aimed to assess the frequency of *Staphylococcus* spp. and their related virulence factors in foodstuffs in Isfahan, Iran.

**Methods:**

Overall, 139 foodstuff samples, collected from Isfahan City (center of Iran) from Sep 2015 to Oct 2016, were processed for the presence of *Staphylococcus* spp. using standard bacteriological procedures and sequence analysis of 16S rRNA gene. Antimicrobial susceptibilities and prevalence of *mecA* and toxin-encoded genes (*sea*, *seb*, *sed*, *see* and *tsst_1_*) were tested for all of the *Staphylococcal* isolates.

**Results:**

Forty-four Gram-positive cocci were recovered from 139 dairy and meat samples*.* The most prevalent species were *S. vitulinus* 25.0% (11/44) and *S. aureus* 20.5% (9/44); respectively. The most prevalent antimicrobial resistance was noted towards penicillin, cefoxitin and tetracycline. The *sec*, *sea*, *see* and *tsst1* genes were found in 19%, 9.5%, 3.5%, and 3.5% of the isolates, respectively.

**Conclusion:**

Numerous virulence factors were detected in different *Staphylococcus* spp. isolated from foodstuffs, more attention should be paid to the presence of the bacteria. Proper hygienic and management practices should be considered in order to increase food safety and prevent extra treatment costs.

## Introduction

Food-borne diseases (FBD) are defined by WHO as “diseases of infectious or toxic nature or thought to be caused by food or water consumption” (1). Symptoms vary widely, depending on the etiological agents with diarrhea and vomiting as the most common symptoms (2). Among FBDs, food-borne infections are caused by many microbial pathogens that can contaminate foods. Food-borne poisoning is caused by poisonous chemicals, microbial toxin, or other harmful substances that are present in food (3). On the whole, FBDs are responsible for nearly 76 million illnesses, 325,000 hospitalizations, and 5,200 deaths each year in the world. They account for 14 million illnesses, 60,000 hospitalizations, and 1,800 deaths annually (4).

*Staphylococcus* strains produce toxins resulting in symptoms ranging from gastrointestinal disorders to paralysis and death (5). Although staphylococcal contamination can be readily eradicated by heat treatment of food, it remains a major cause of FBD (5). *Staphylococcus aureus* is able to grow in a wide range of temperatures, pH and NaCl concentrations (5, 6). Their resilience in being able to grow under a wide range of temperature, pH and osmolality justifies how *S. aureus* can be readily present in foodstuffs especially those often manipulated during processing, e.g. sausages, salads, cream-filled bakery items, sandwich equipment and dairy products (5).

Staphylococcal food poisoning (SFP), widely assigned to toxigenic *Staphylococcus,* takes place following ingestion of at least 1.0 μg of enterotoxin in food (7). The most remarkable virulence factors associated with staphylococci are the heat-stable enterotoxins (SEs) secreted by certain strains. The *Staphylococcus* Enterotoxins (SEs) are divided into five classical serological types: *Staphylococcus* Enterotoxin A (SEA), *Staphylococcus* Enterotoxin B (SEB), *Staphylococcus* Enterotoxin C (SEC), *Staphylococcus* Enterotoxin D (SED) and *Staphylococcus* Enterotoxin E (SEE). However, recently other enterotoxins were reported in the literature, including SEG, SHE, SEI, SER, SES, SET and the enterotoxin-like proteins such as SElK, SElN, SElO, SE1P, SE1Q and SElU (7, 8). Reliable detection of SE genes serves a twofold function. Firstly, it aids genotyping of the coagulase-positive staphylococci (CPS) for epidemiological studies. Secondly, it provides an assessment of the possible occurrence of SE genes in strains of Coagulase-Negative Staphylococci (CNS) often used as starters in food fermentation (8, 9).

The aim of the present study was to investigate the prevalence of *Staphylococcus* isolates and the toxin *sea, seb, sed, see* and *tsst1* genes as well as their antimicrobial susceptibility patterns in isolates from a variety of food sources collected in Isfahan, Iran.

## Materials and Methods

### Sampling and identification

In a previous study (10), we had sampled 55 confectionaries for isolation and identification of *Staphylococcus* species. Forty isolates were recovered samples that belonged to different species (30% (12/40) *S. aureus*, 17/5% (7/40) *S. succinus* subsp*. casei*, 15 (6/40) *S. warneri*, 7.5% (3/40) *S. carnosus*, 5% (2/40) *S. pasteuri*, 5% (2/40) *S. vitulinus*, 5% (2/40) *S. sciuri*. 5% (2/40) *S. epidermidis*, 2.5% (1/40) *S. succinus* subsp*. succinus*, 2.5% (1/40) *S. lugdonensis*, 2.5% (1/40) *S. saprophyticus* and 2.5% (1/40) *S. gallinarum*). In the present study, from Sep 2015 to Oct 2016, 139 other foodstuff samples including dairy products (cheese, cottage and yogurt) and meat products (sausages, and hamburgers) belonging to 18 different brands were collected from 29 stores in various parts of Isfahan city (center of Iran). The samples were then processed within 12h of their collection in microbiology laboratory of Isfahan Infection Diseases and Tropical Medicine Research Center. Isolation of *Staphylococcus* species was performed as written in our prior study (10).

### Antimicrobial susceptibility test

The Clinical and Laboratory Standard Institute (CLSI, 2017) reference method for disk diffusion was used for antimicrobial susceptibility test of all *Staphylococcus* isolates (11). The following antibiotics were tested using the standard antibiotic disks (Mast Group, UK) (concentrations are expressed in μg ml^−1^): penicillin (10 units), cefoxitin (30), gentamicin (10), tetracycline (30), ciprofloxacin (5), clindamycin (2), trimethoprimsulfamethoxazole (1.25/23.75), chloramphenicol (30), rifampin (5), linezolid (30), levofloxacin (5), and erythromycin (15).

### DNA Extraction for Molecular Assays

DNA of all isolates was extracted using boiling method (12). In brief, a few colonies of each isolate were added to 100 μl of TE buffer (10 mM Tris, 1 mM EDTA, pH 7.8) and boiled for 15 min at 100 °C. After centrifugation at 9,000 × g for 5 min at 4 °C, supernatant fluid was transferred into a new sterile tube and stored at −20 °C.

### Molecular Identification of Staphylococcus Species

The *Staphylococcus* spp. isolated from dairy and meat products and identified phenotypically according to conventional methods were further analyzed to the species level by sequence analysis of 16S rRNA gene (13). The sequence data were analysis by the Clustal W v2.0 software and Gen-Bank database (14).

### Identification of mecA gene

A PCR reaction was carried out for the amplification of the 310 bp fragment of *mecA* gene using primers as was shown in Table 1. The PCR reaction mixture (25μL) contained: 4μL of DNA template, 2.5 μL of PCR buffer (×10), 0.75 μL MgCl_2_ (50 mM), 0.5 μL of dNTPs (10 mM), 1 μL of each primers (2 μL totally), 0.25 μL of Ex-Taq DNA polymerase (5u/μL) and 15 μL distilled water. The PCR conditions were as follows: Initial denaturation at 94 °C for 5 min, 30 cycles of denaturation at 94 °C for 30 sec, annealing at 55 °C for 30 sec and extention at 72 °C for 30 sec, and final extension at 72 °C for 7 min (15).

**Table 1:** Primers for Amplification of toxin encoding and 16S rRNA genes of *Staphylococcus* isolates

***Gene***	***Primer***	***Oligonucleotida sequence (5–3)***	***Amplicon size(bp)***
*sea*	SEA-1 SEA-2	TTGGAAACGGTTAAAACGAA GAACCTTCCCATCAAAAACA	120
*seb*	SEB-1 SEB-2	GGTACTCTATAAGTGCCTGC TTCGCATCAAACTGACAAACG	475
*sec*	SEC-1 SEC-2	AGAACTAGACATAAAAGCTAGG TCAAAATCGGATTAACATTATCC	267
*sed*	SED-1 SED-2	TTTGGTAATATCTCCTTTAAACG CTATATCTTATAGGGTAAACATC	309
*see*	SEE-1 SEE-2	CCTATAGATAAAGTTAAAACAAGC TAACTTACCGTGGACCCTTC	173
*Tsst1*	TSST1-1 TSST1-2	ATGGCAGCATCAGCTTGATA TTTCCAATAACCACCCGTTT	350
*mecA*	MECA-1 MECA-2	GTAGAAATGACTGAACGTCCGATAA CCAATTCCACATTGTTTCGGTCTAA	310
*16S rRNA*	27F 515R	AGAGTTTGATCMTGGCTCAG TTACCGCGGCKGCTGGCAC	530

### Identification of TSST1 and enterotoxins Genes

All *Staphylococcus* isolates were tested for toxin genes by two specific multiplex PCRs: (I) 120, 309 and 350 bp fragment of the *sea, sed*, and *tsst1* genes; respectively (16), (II) 475, 267, 173 bp fragment of *seb, sec*, and *see* genes (17). Specific primers for amplifying the enterotoxin-encoding genes and *tsst1* gene by PCR are shown in Table 1.

## Results

### Isolation and characterization of the isolates

Overall, 44 Gram-positive cocci with a positive catalase reaction were recovered from the 139 dairy and meat samples initially collected from diverse regions in Isfahan, Iran. 72.73% and 27.27% of *Staphylococcus* spp. were isolated from meat products and dairy products samples, respectively. Based on 16S rRNA gene sequence analysis, these *Staphylococcus* spp. isolates belonged to 11 validated species: *S. vitulinus* 25% (11/44), *S. aureus* 20% (9/44), *S. warneri* 9% (4/44), *S. epidermidis* 9% (4/44), *S. equorum* 9% (4/44), *S. succinus* subsp*. casei* 6.8% (3/44), *S. saprophyticus* 6.8% (3/44), *S. pasteuri* 4.5% (2/44), *S. gallinarum* 4.5% (2/44), *S. xylosus* 2.3% (1/44) and *S. simulans* 2.3% (1/44).

### Antibiotic susceptibility pattern

The results of antibiotic susceptibility test revealed that, all *Staphylococcus* spp. isolates were susceptible to rifampicin, levofloxacin, ciprofloxacin and gentamicin.

**Penicillin**: The prevalent resistance towards penicillin was seen in *S. pasteuri* (100%), *S. succinus sub succinus* (100%), *S. gallinarum* (100%) and *S. xylosus* (100%). *S. epidermidis* (83.3%), *S. warneri* (80%), *S. aureus* (76.2%), *S. equorum* subsp*. linens* (75%), *S. saprophyticus* (75%), *S. sciuri* subsp*. sciuri* (50%), *S. succinus* subsp*. casei* (50%) and *S. vitulinus* (7.7%) were next in rank, respectively.

**Tetracycline**: The most prevalent tetracycline resistance was seen in *S. saprophyticus* (50%), *S. warneri* (50%), *S. equorum* subsp*. linens* (25%), *S. pasteuri* (25%), *S. succinus* subsp*. casei* (20%), *S. epidermidis*(16.7 %), *S. vitulinus* (14.4%), and *S. aureus* (14.3%) isolates, respectively.

**Cefoxitin**: *S. sciuri* subsp*. sciuri* (100%), *S. succinus sub succinus* (100%), *S. epidermidis* (50%), *S. equorum* subsp*. linens* (25%), *S. succinus* subsp*. casei* (20%), *S. warneri* (20%) and *S. vitulinus* (7.7%) had highest resistance for cefoxitin, respectively.

**Erythromycin**: *S. epidermidis* (50%), *S. equorum* subsp*. linens* (25%), *S. saprophyticus* (25%), *S. warneri* (20%), *S. succinus* subsp*. casei* (20%) and *S. aureus* (4.8%) had the most prevalence of resistance, respectively.

**Chloramphenicol:** Resistance for chloramphenicol was only seen in *S. saprophyticus* (50%).

**Linezolid:** *S. pasteuri* (25%) and *S. aureus* (4.8%) were non-susceptible to linezolid.

**Clindamycin:** 20% of *S. warneri isolates* and 7.7% of *S. vitulinus* isolates showed resistance for clindamycin.

**Trimethoprim & Sulfamethoxazole:** The most prevalent Trimethoprim & Sulfamethoxazole resistance was seen in *S. epidermidis* (33.3%), *S. warneri* (30%), *S. equorum* subsp*. linens* (25%), and *S. succinus* subsp*. casei* (10%), respectively.

### Screening of mecA gene

Prevalence of *mecA* gene in all isolates was 38.5% in *S. vitulinus*, 33.3% in *S. epidermidis*, 30% in *S. warneri*, 25% in *S. equorum*, 10% in *S. succinus* subsp*. casei* and 9.5 % in *S. aureus*. Others *Staphylococcus* spp. isolates were negative.

### Screening of TSST-1 and enterotoxins Genes

All isolates were screened for enterotoxin production and *tsst-1* genses by specific PCR (Table 1). *sea* gene was found in 8 (9.5%) of the 84 isolates of *Staphylococcus* spp. The *sec* gene was found in 16 (19%) of the 84 isolates of *Staphylococcus* spp. Three (3.5%) of the 84 isolates of *Staphylococcus* spp. were positive for *see* gene. Identification of methicillin-resistant strains of staphylococci by polymerase chain reaction. of *Staphylococcus* spp. Among all isolates, *tsst-1* gene was only found in *S. aureus* (9.5%) and *S. saprophyticus* (25%). The *seb* and *sed* genes were not found in any of the *Staphylococcus* spp. isolates (Table 2).

**Table 2:** Identification of enterotoxin-encoding genes and TSST-1 of *Staphylococcus* isolates in foodstuff product by molecular methods

***Specie***	***No. of Isolates***	***sea (%)***	***seb (%)***	***sec (%)***	***sed (%)***	***see (%)***	***tsst_1_ (%)***
*S. warneri*	10	-	-	30	-	-	-
*S. succinus sub succinus*	1	-	-	-	-	-	-
*S. succinus sub casei*	10	10	-	-	-	-	-
*S. vitulinus*	13	-	-	7.7	-	7.7	-
*S. pasteuri*	4	-	-	25	-	-	-
*S. aureus*	21	23.8	-	28.6	-	4.8	9.5
*S. lugdunensis*	1	100	-	-	-	-	-
*S. saprophyticus*	4	-	-	25	-	-	25
*S. epidermidis*	6	-	-	17.7	-	-	-
*S. gallinarum*	3	-	-	33.3	-	-	-
*S. carnosus*	3	-	-	33.3	-	-	-
*S. equorum*	4	-	-	25	-	25	-
*S. xylosus*	1	-	-	-	-	-	-
*S. simulans*	1	-	-	-	-	-	-
*S. sciuri* subsp*. sciuri*	2	50	-	-	-	-	-

## Discussion

SFP is an intoxication that results from the consumption of foods containing sufficient amounts of one or several of the preformed SE (18, 19). Foods frequently contaminated with SE include meat and meat products, poultry and egg products, milk and dairy products, bakeries, and sandwich fillings (2, 20). The disease is usually self-limiting and typically resolves within 24–48 h after the onset. However, in some cases, the affliction can be severe enough to lead to hospitalization, particularly in cases involving the elderly, infants, and those with frail health (2, 21).

Identification of *Staphylococcus* species is quite significant for epidemiological investigations as well as to assess virulence factors such as enterotoxin production and the development of specific management practices to prevent SFPs caused by CNSs (22). V1 region is the best region to differentiate between *S. aureus* and CNSs (23). In this study, from 139 foodstuff samples collected, 44 (31.6%) isolates belonged to 11 different species and subspecies of *Staphylococcus*. Prevalence of *S. aureus* was 20/5%, which was lower than those reported by others (19% to 48.7% range) (24–28). Molecular analysis showed that 27.4% (23/84) of the isolates carried one or more SE genes. Four SE genotypes were detected. The most commonly detected SE genes were *sec*, *sea*, *see* and *tsst_1_* with 19, 9.5, 3.5 and 3.5 percent occurrence, respectively. The frequent detection of SE genes among *Staphylococcus* spp. taken from different sources has already been demonstrated by various research groups (28–30). Our results are in agreement with these studies that demonstrated the enterotoxigenicity of more than 40% of the isolates of *S. aureus* collected from various food products. In contrary to our finding, in China, the most frequently seen SE genes was *sea* (86.5%, 45/52). Four SE gene profiles were observed, including *sea* (86.5%), *sec*-*she* (5.8%), *seb* (n=3.8%), and *seg-sei* (n=3.8%) (31). This discrepancy in frequency rate of SE genes suggests that probably some factors such as differences in food products, diagnostic methods and geographical distribution, may be effective in the variation of results.

*Staphylococcus aureus* has been known to be frequently resistant to antibiotic therapy due to their capacity to produce an exopolysaccharide barrier and a substantial quantity of antibiotic neutralizing enzyme that limits the antibiotic action (9, 32). Overall, 27% of the isolates were sensitive to all the tested antibiotics and 45% of the strains were shown to be intermediate (according to CLSI, 2017) and resistant to at least 4 antibiotics (data not shown). The isolates collected from dairy products were demonstrated to be most sensitive to the tested antibiotics (80%). No resistance to rifampicin, levofloxacin, ciprofloxacin, gentamicin and clindamycin was observed, while a relatively small percentage of the isolates demonstrated resistance to cefoxitin (14.3%), erythromycin (10.8%), and chloramphenicol (2.4%).

Similar to our finding, the most remarkable resistance was reported against penicillin (96.2%, 50/52) and followed by resistance to tetracycline (28.8%, 15/52) (31). Furthermore, a high level of penicillin-resistance (71.4%) was reported among *S. aureus* isolates from food samples (33).

In addition, in Brazil, where 227 CoNS isolates were recovered from 35 cheese samples, antibiotic susceptibility pattern of isolates showed a high level of resistance against penicillin (78.5%), and erythromycin (67.8%) which is inconsistent with our results (34).

## Conclusion

Characterization of *Staphylococcus* species and enterotoxin-encoding genes is crucial for epidemiological investigations. Detection of enterotoxin-encoding genes and antibiotic resistance in *staphylococcal* spp. isolated from food indicated that food may represent a potential health risk.

## Ethical considerations

Ethical issues (Including plagiarism, informed consent, misconduct, data fabrication and/or falsification, double publication and/or submission, redundancy, etc.) have been completely observed by the authors.
